# Development and evaluation of clinically effective “APOE Genotyping PCR‐based assay kit” in Alzheimer’s Disease patients

**DOI:** 10.1002/alz.095484

**Published:** 2025-01-09

**Authors:** Nozomi Kojima, Kozue Matsuda, Hikaru Hattori, Motonari Daitho, Tamao Tsukie, Ai Obinata, Akinori Miyashita, Takeshi Ikeuchi

**Affiliations:** ^1^ SYSMEX Corporation, Kobe, Hyogo Japan; ^2^ Brain Research Institute, Niigata University, Niigata, Niigata Japan

## Abstract

**Background:**

The Alzheimer’s disease treatment drug “Lecanemab” has been approved in the US, Japan, and China, and administration has begun in clinical treatment. Other anti‐Aβ antibody drugs (e.g., Donanemab) with the same mechanism of action are also being developed. Adverse effect of Amyloid Related Imaging Abnormalities (ARIA) has been reported and the incidence of ARIA varies by genotype (van Dyck et al, N Engl J Med 2023). In the US and Japan, it is recommended that APOE genotyping tests should be performed more than protein phenotyping for predicting the risk of ARIA, and there is a strong clinical need for quality‐assured test.

We have developed a rapid, high‐throughput, quality‐controlled PCR testing method that can achieve low costs that will help reduce medical costs.

**Method:**

This assay detects two SNPs in two sites including the GC‐rich region associated with the risk of ARIA, which previously required two PCR assays. By designing of primer/probe sequences and Tm value control and PCR cycle conditions, all six APOE genotypes could be classified with one assay. To evaluate the clinical utility of this assay, we conducted an evaluation using DNA samples derived from whole blood specimens in which APOE genotype was determined.

**Result:**

In this APOE genotyping assay, the number of assays was reduced from 2 to 1, and results could be reported in approximately 100 minutes per PCR run by a simple and low‐cost PCR‐based assay. In the clinical evaluation, a total of 130 samples were tested, and the APOE genotype concordance rate for all 6 types (ε2/ε2, ε2/ε3, ε2/ε4, ε3/ε3, ε3/ε4, ε4/ε4) was 100% and compared to the reference method, Sanger sequencing.

**Conclusion:**

The newly developed assay was confirmed that it is a low‐cost and high‐throughput PCR‐based assay and can accurately determine the six genotypes. In the future, it is expected that this assay will be widely used to determine the risk of ARIA before administering anti‐Aβ antibody drugs. We will also report on sample quality and points to consider required for APOE genotyping.

